# Migraine with aura: less control over pain and fragrances?

**DOI:** 10.1186/s10194-023-01592-3

**Published:** 2023-05-17

**Authors:** Coralie Mignot, Vanda Faria, Thomas Hummel, Marie Frost, Christoph M. Michel, Gudrun Gossrau, Antje Haehner

**Affiliations:** 1 Department of Otorhinolaryngology, Faculty of Medicine Carl Gustav Carus, Smell & Taste Clinic, Technische Universität Dresden, Fetscherstraße 74, 01307 Dresden, Germany; 2 Department of Psychology, Uppsala University, 752 37 Uppsala, Sweden; 3 Brain and Eye Pain Imaging Lab, Pain and Affective Neuroscience Center, Boston Children’s Hospital, Harvard Medical School, Boston, MA MA 02115 USA; 4 Department of Anesthesiology, Critical Care and Pain Medicine, Boston Children’s Hospital, Harvard Medical School, Boston, MA MA 02115 USA; 5 Comprehensive Pain Center, University Hospital and Faculty of Medicine Carl Gustav Carus, TU Dresden, Fetscherstraße 74, 01307 Dresden, Germany; 6 Department of Basic Neurosciences, University of Geneva, CH-1211 Geneva 4, Switzerland; 7 CIBM Center for Biomedical Imaging, 1015 Lausanne, Switzerland

**Keywords:** Migraine, Aura, Olfaction, Trigeminal, EEG

## Abstract

**Background:**

Accumulating data emphasizes the importance of olfaction in migraine pathophysiology. However, there are only a few studies evaluating how the migraine brain processes olfactory stimulation, and virtually no studies comparing patients with and without aura in this context.

**Methods:**

This cross-sectional study recorded event-related potentials from 64 electrodes during a pure olfactory or pure trigeminal stimulus in females with episodic migraine with aura (*n* = 13) and without aura (*n* = 15), to characterize the central nervous processing of these intranasal stimuli. Patients were tested in interictal state only. Data were analyzed in the time domain and in the time–frequency domain. Source reconstruction analysis was also performed.

**Results:**

Patients with aura had higher event-related potentials amplitudes for left-sided trigeminal and left-sided olfactory stimulations, and higher neural activity for right-sided trigeminal stimulation in brain areas related to trigeminal and visual processing. Following olfactory stimulations patients with aura displayed decreased neural activity in secondary olfactory structures compared to patients without aura. Oscillations in the low frequency bands (< 8 Hz) differed between patient groups.

**Conclusions:**

Altogether this may reflect hypersensitivity to nociceptive stimuli in patients with aura relative to patients without aura. Patients with aura have a bigger deficit in engaging secondary olfactory-related structures, possibly leading to distorted attention and judgements towards odors. The cerebral overlap between trigeminal nociception and olfaction might explain these deficits.

**Supplementary Information:**

The online version contains supplementary material available at 10.1186/s10194-023-01592-3.

## Background

Among neurological disorders, migraine is the leading cause of disability and is associated with increased sensitivity to light, sound, touch, and smell. Migraine attacks are more common in women than in men, last longer and are more disabling [1–4]. Accumulating data emphasizes the importance of olfaction in migraine pathophysiology. Osmophobia (aversion and intolerance to odors) in particular, affects up to 90% of the migraine patients [5–7] and is also experienced interictally, i.e. apart from migraine attacks [8]. Moreover, odors, especially perfumes and cleaning products, can trigger headaches in around 45% of these patients [5, 6, 9], and this seems to be specific to migraine headaches [10–13].

Although the olfactory system has central role in migraine, there are to date only few imaging studies evaluating how the brain of people with migraine process olfactory [14–17] or intranasal trigeminal stimulation [15, 18–20]. Migraine patients are subjected to specific cerebral states, as shown by an enhanced deep cerebral activity for these patients in the amygdala, the insula, the rostral pons, the piriform cortex, the temporal pole and the antero-superior temporal gyrus in the interictal state [14], during spontaneous migraine attacks [17], in resting-state [14] or in response to odors [14, 17]. Decreased cortical activity during odor processing [14–16], together with increased cortical activity during trigeminal processing was also reported [15]. The prefrontal cortex and the rostral anterior cingulate cortex, both known to be involved in pain control, showed abnormal activity after intranasal trigeminal stimulation in migraine patients [18].

Whether these results are confounded by the inclusion of patients with aura (MWA) and without aura (MWoA) remains unknown. Yet, several studies point to differences between migraine patients with and without aura in terms of heritability [21], association with ischemic stroke and depression [21, 22], alteration in brain structure [21] and function (especially in the form of cortical hyperexcitability in MWA) [21, 23], and cerebral blood flow changes during attacks [21]. This suggests that MWA and MWoA could be considered as two entities [24] and should be investigated separately [23].

Moreover, odors have been found to be more offensive in MWA than MWoA [7]. Given that most odorants commonly activate both trigeminal and olfactory neurons [25], it is important to understand whether these groups of patients show a differential olfactory brain processing to these two types of stimulation.

Thus, the aim of this study was to characterize, for the first time, the central nervous processing of olfactory and nasal trigeminal stimuli in females with migraine with aura and without aura separately. For this purpose, electroencephalography (EEG) was recorded while patients received a pure olfactory stimulus or a pure trigeminal stimulus, in the left or in the right nostril.

## Methods

### Participants

Twenty-eight women with episodic migraine (monthly days of migraine attacks: mean 4.3 ± Std 2.37), diagnosed according to the International Classification of Headache Disorders, 3rd edition (ICHD III) [26] were recruited for this study (mean age = 35 ± Std 9.8 years, range 21–51 years): 13 MWA (mean age = 32.5 ± Std 8.3 years, range 22–44 years) and 15 MWoA (mean age = 37.1 ± Std 10.6 years, range 21–51 years). The study sample size was based on similar experiments involving EEG assessments of odor stimulations using time domain, time–frequency and source reconstruction analyzes (from 10 to 23 participants per condition) [15, 27–30]. Since women are three to four times more affected by migraine than men, and women outperform men in detecting, discriminating and identifying olfactory cues [31, 32] our study included only women. Patients were tested during the interictal phase only. The interictal phase was defined as a free of migraine-specific symptoms period (including fatigue, concentration disturbances) after a migraine attack of at least 48 consecutive hours. This means that the patients were included in the study only if they did not have a migraine attack 24 h before and 24 h after the investigation. They were recruited at the Headache Clinic at the University Pain Center at TU Dresden between October 2019 and December 2020. The exclusion criteria were the following: pregnancy, major chronic disease, olfactory loss, relevant sinunasal diseases (i.e., chronic rhinosinusitis, allergic rhinitis, nasal polyps), asthma, acute mental disorders, attentional dysfunction, insufficient communication skills, and smoking. Patients were all right-handed. For details regarding participants demographics and clinical characteristics see Table 1. All participants provided written informed consent. Data was collected in accordance with the declaration of Helsinki related to human research and with the Declaration of the World Medical Association (www.wma.net). The protocol was approved by the Ethics Committee of the TU Dresden (GVOEK) under the application number EK 58,022,015. For their participation, the subjects received financial compensation. The datasets used and/or analysed during the current study are available from the corresponding author on reasonable request.

**Table 1:** Population description

Population description	MWA *n* = 13	MWoA *n* = 15
Age	32.5 ± Std 8.3 years, range 22–44 years	37.1 ± Std 10.6 years, range 21–51 years
Disease duration (years)	14.8 ± Std 8.6	13.1 ± Std 9.7
Frequency of migraine days in the past month	4.4 ± Std 2	4.2 ± Std 2.7
Duration of attacks (hours) related to the frequency of migraine attacks in the past month (regardless whether acute medication was taken or not)	4.5 ± Std 3.1	14.3 ± Std 21.8 (2 patients above 60 h)
Mean intensity of migraine attacks in the past month (from 0 = not intense to 10 = extremely intense)	4.2 ± Std 1.5	5 ± Std 2
Osmophobia (yes/no)	Q1 “Are you sensitive to scents before/during the migraine attack”: 6 yes/6 no/1 missing Q2 “Can odors trigger a migraine attack”: 3 yes/9 no/1 missing	Q1 ““Are you sensitive to scents before/during the migraine attack”: 7 yes/7 no/1 missing Q2 ““Can odors trigger a migraine attack”: 1 yes/13 no/1 missing
Symptoms during migraine attacks associated with the sensitivity to odors (yes/no)	3 yes/7 no/3 missing	5 yes/10 no

Description of migraine patients with aura (MWA) or without (MWoA)

### Stimulations

Stimuli were presented using a computer-controlled olfactometer (OM6b, Burghart, Holms, Germany) that allows for the presentation of odorants in a constant airflow of 6L/min, humidified air of controlled temperature (36 °C, relative humidity 80%). Stimuli were presented by means of a Teflon tube (8 cm length, 4 mm inner diameter) inserted into the nostril (right or left) for around 1 cm in order to reach beyond the nasal valve region. Twenty stimuli of each condition (olfactory: chocolate (PG93157, Fragrance Resources GmbH, Germany) 50% v/v diluted in an odorless airflow, and trigeminal stimulation: CO_2_, 45% v/v) were presented (200 ms stimulus duration) with a 16 s average inter-stimulus interval (range: 14-18 s).

### Procedure

The procedure was divided in two main sessions of one hour and a half each separated by a break of 5 to 10 min. One session was dedicated to psychophysical testing and the other consisted in EEG measurements.

The EEG experiment took place in an air-conditioned room set at a temperature of 20–22 °C. During the EEG experiment itself, the patients were subjected to four sessions of 5 min each in which 20 stimuli of one of the stimulation types were sent through a tubing to one nostril. The session order was not counterbalanced due to the fact that migraine patients are very sensitive and the CO_2_ stimulation may disturb the following assessment. Thus, the order was always « chocolate» followed by CO_2_ in the left nostril and then in the right nostril (chocolate left, CO_2_ left, chocolate right, CO_2_ right). Nostrils were stimulated separately to avoid the airflow to reach the olfactory epithelium at different timings in the case of birhinal stimulation. This had also the advantage to minimize habituation. Patients also performed a tracking task (following with the computer mouse a square moving on the computer screen) to maintain their attention throughout the EEG recording.

### Psychophysical chemosensory testing

The olfactory performance of the patients was assessed using the Sniffin’ Sticks test [32, 33], a standardized test subdivided in three tasks namely Threshold (the ability to detect a smell), Discrimination (the ability to differentiate smells) and Identification (the ability to label smells). The threshold test consists of 16 triplets of increasing odor concentrations. At each trial, the patient has to choose the stick among three that contains a smell, the two others being odorless. This is a single-staircase, three alternatives forced-choice procedure. The order of triplets’ presentation (increasing the odor concentration or decreasing it) is adapted according to the false and right answers until the test reaches seven turning points, and a mean threshold is calculated with the four last turning points. The discrimination test consists also of 16 triplets of sticks. In this case however, all the sticks contain a high concentration scent. At each trial, amongst the three sticks, two odors are identical and the third one is different. The aim is to determine the odd one. A discrimination score corresponding to the sum of the correct answers is established. The identification test includes also 16 sticks filled with high concentration odors, and they are presented one by one, each of them with an images booklet. The aim for the patient is to point out the item corresponding to the odor among four alternatives. The identification score corresponds to the sum of the correct answers. The sum of the scores from the three subtests resulted in the TDI score with a maximum of 48 points. A TDI score of 30.5 points or more suggests normosmia, a score between 16.5 and 30 points indicates reduced olfactory function in terms of hyposmia, and a score of less than 16.5 points implies anosmia. The trigeminal sensitivity of the patients was assessed using a lateralization test with a trigeminal eucalyptus smell. In this task, two identical airflows were applied to both nostrils using a handheld “squeezing device” which releases the same amount of air simultaneously to the left and right nostrils. One side received the target odorant, while the other side received odorless air. The sides of the odorant stimulation were changed in pseudo-randomized order. If the odorant has a trigeminal component, and if the patient has a good trigeminal sensitivity, the success rate in detecting the stimulated nostril increases significantly. See the details elsewhere [34, 35].

### EEG recording

Sixty-four Ag–AgCl pin-type active electrodes recording the scalp potentials were mounted on a cap using gel, according to the 10–20 system. Eight Ag–AgCl flat-type electrodes were used, two were placed on the earlobes and two on the mastoids in case we wanted to test another offline referencing, and four to identify vertical and horizontal eye-blinks in the signal (two above the lateral extremity of the eyebrows and two on the lateral side of the eyes). The recording was performed with a Biosemi bioamplifier (BioSemi, Amsterdam, Netherlands) and its associated software Actiview. The sampling rate was 512 Hz. Impedance was kept below 25 kOhm.

### EEG pre-processing and processing steps

#### General steps

The preprocessing steps were performed using Letswave 7 software (https://letswave.cn/) and EEGLAB 2020 (La Jolla, CA, USA).

The following steps apply to each participant and condition. A Butterworth filter was applied with high-pass at 0.1 Hz and low-pass at 30 Hz. Epochs were segmented from 500 ms pre-stimulus to 2000 ms post-stimulus. A baseline correction was applied from -500 ms to 0 ms (relative to the stimulus onset). The signal was visually inspected to determine which epochs have to be rejected (lead movements, muscular artefacts) regardless of the eye-blinks artefacts that were corrected later on. Bad electrode interpolation occurred when necessary using spherical spline interpolation. The signal was offline referenced to the common average. Independent Component Analysis (ICA) was performed to remove eye-blink components from the data (EEGLAB 2020, La Jolla, CA, USA) with “runica” as algorithm, using a PCA (principal component analysis) step and a fixed number of 30 components. Components considered as eye-blink artefacts were discarded on the basis of: 1) frontal distribution of the ICA weights on the scalp map, 2) large amplitude dynamics randomly distributed across the trials, 3) time-course mainly affected by characteristic short and large monopolar potentials, 4) smoothly decreasing power spectrum. After the ICA, epochs were checked again and those whose amplitude exceeded 80 µV were excluded, as well as remaining eye-movements occurring in the -500 to 1500 ms post-stimulus range. The remaining epochs were averaged for each stimulation, nostril side and participant separately. For detailed information regarding the number of epochs averaged in each group and condition, and the number of patients with missing data, see Additional files 1 and 3.

#### Frequency domain pre-processing steps

The time–frequency analysis (TFA) was applied on the previously preprocessed and accepted single epochs in two more steps: a Continuous Wavelet Transform (CWT) in the 0.3-30 Hz bandwidth in 100 steps, and a supplementary baseline correction (substraction method) from -400 ms to -100 ms relative to the stimulus onset, before averaging at the group level. For more information about this procedure, see Huart and colleagues’ paper [27]. While the time-domain averaging gives an idea of the phase-locked activity of the brain response to chocolate or CO_2_ sent in the left or in the right nostril, the analysis using CWT was used in order to reflect the non-phase locked activity.

#### EEG source imaging

EEG source imaging was implemented in the Cartool 3.91 (6638) software (https://sites.google.com/site/cartoolcommunity/) using the signal from the 64 electrodes. Full head and segmented brain MNI templates from 152 healthy subjects with 114 regions of interest included in the AAL atlas, an electrodes coordinate file adapted to the Biosemi system with 64 channels and coregistered to the MRI, were used for the source reconstruction algorithm implementation. All these files and the solution points file (4888 solution points) were provided by Cartool. The lead field was computed using the LAURA source localization algorithm [36] from the solution points and electrodes in a semi-automated way guided by Cartool, using the LSMAC (Locally Spherical Model with Anatomical Constraints) on the individual means of each condition separately over the whole ERP time window.

### Event-related potentials (ERP) assessment

The presence, amplitude and latencies of the ERP were assessed on the Pz electrode only. The literature on olfactory and trigeminally elicited cerebral responses usually reports midline positions as the best recording sites [27, 30, 37–43]. However, it has been shown that N1 and P2 components for trigeminal stimuli have maximum amplitudes over Cz [43] and over Pz when it comes to olfactory ERP [39, 41, 43]. As the SNR is usually better for trigeminal ERP than olfactory ERP [39], Pz seems to be a good compromise. The presence of the ERP was estimated considering the shape and latency of the different peaks: a small positivity (P1) in the range of 200-320 ms post-stimulus followed by a medium negativity (N1) in the 200-700 ms post-stimulus range, a large positivity (P2) in the 300-800 ms post-stimulus range and a large positivity (P3) in the 700-1100 ms range. In few occasions (27 latency measurements out of 316), the measurements of latencies were out of these boundaries (for more information, see Additional file 2) but were still included in the analyses, as the previous ranges are defined for a healthy population but that the present study is focused on general migraine patients. The latency ranges and the peak designation were defined according to the literature on chemosensory ERPs [37, 39, 44]. There is no consensus on the terminology of the different ERP peaks in the chemosensory research domain: in some studies P2 and P3 are considered as a unique component; when divided as here, P2 usually refers to the P3a described in other sensory modalities while P3 is the late positive component [44] equivalent to P3b in other sensory modalities. The latencies of the different peaks (P1, N1, P2, P3), the peaks’ relative values (relative to the baseline) and peak-to-peak amplitudes were measured heuristically by one observer (P1, N1, P2, P3, N1P2, N1P3).

### Data analyses

#### Classical group comparisons

The features of the ERP peaks were compared between MWA and MWoA using two-tailed unpaired t-test or a Mann–Whitney test (according to their *p*-value at the Shapiro–Wilk test of normality: *P* < 0.05 would lead to the Mann–Whitney test).

A two-tailed unpaired t-test was applied on the time–frequency analysis maps, comparing MWA and MWoA (MWA being the reference subset).

#### Receiver operating characteristic curves and discrimination performance

The ability to differentiate MWA from MWoA based on each of their ERP features (amplitudes of N1, P2, P1N1, and N1P2) and each of the stimulus conditions was assessed using Receiver Operating Characteristic (ROC) curves. A ROC curve is represented by the specificity (true positive rate) plotted against sensitivity (false positive rate). The area under the curve (AUC) was calculated and indicated good discrimination performance (when AUC is close to 0 or 1) or bad discrimination performance (when AUC is close to 0.5) of each feature.

#### Source imaging

An unpaired t-test was applied via Cartool on the results of the inverse solution to compare source signal between MWA and MWoA. The whole epoch time course (pre- and post-stimulus) was considered and the t-test was applied on each time-frame, with a level normalization using the mean Gfp. The results of this t-test are reported for a minimum of significant differences on 16 consecutive time-frames (~ 31 ms).

#### Statistical analyses

For all the tests, the normality of the data was checked using the Shapiro–Wilk test. Whenever a deviation from normality was detected, the corresponding non-parametric tests were used. A repeated measures ANOVA was performed for each peak individually (P1, N1, P2, P3) using the nostril side (right or left) and stimulation type (chocolate or CO2) as repeated measures factors and the aura status as between subject factor. T-tests were performed as post hoc tests. In the eventuality of such tests, the *p*-values reported were Bonferroni corrected. *P* values and degrees of freedom are reported. In addition, two-tailed unpaired t-test or Mann–Whitney test were applied on amplitudes and latencies of the ERP peaks. For all tests, a *P* < 0.05 was considered as significant. Some effect sizes are reported, they correspond to Eta squared values for the repeated measures ANOVA, Cohen’s d for the unpaired t-tests and rank biserial correlation for the Mann–Whitney tests. Regarding source imaging, uncorrected findings were considered.

## Results

### Results from psychophysical chemosensory testing

All the patients were considered normosmics as they had similar general olfactory function compared to healthy subjects from published normative data [32] (TDI scores, two tailed Wilcoxon signed rank test, *Z(df 27)* = 155, *P* = 0.279). However, when assessing the individual olfactory subtests, patients had a lower olfactory sensitivity (i.e. higher olfactory thresholds) compared to the normative data (two-tailed one sample t-test *t(27)* = 2.66, *P* = 0.013, effect size = -0.50). No differences were found for olfactory discrimination and identification abilities.

The overall olfactory function, the discrimination and identification performances were not significantly different for MWA and MWoA. However, the MWA had higher thresholds than MWoA, i.e. MWA odor detection ability was worse than in MWoA (Mann–Whitney test, *U* = 36.5, *P* = 0.005, effect size = -0.63).

### Time-domain results

**Fig. 1:**
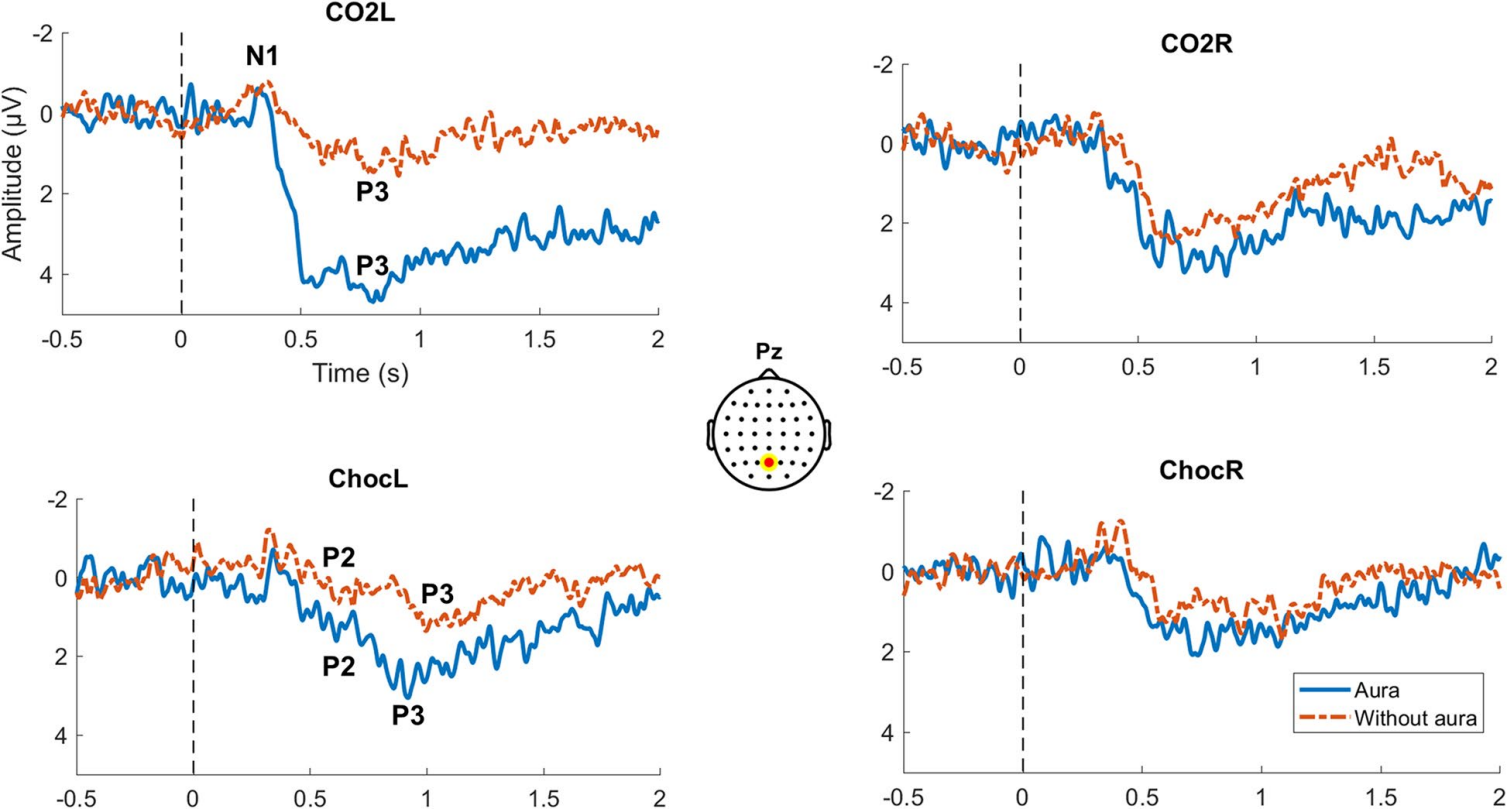
Group level trigeminal and olfactory ERP in MWA and MWoA, in left or right nostrils. The stimulus onset is positioned at 0 s (dotted black line). Signals were taken from the Pz electrode. Bold blue line: MWA; Dotted red line: MWoA. Choc: Chocolate, L: left, R: right. Significant differences in peaks amplitudes between aura and without aura groups were found with CO_2_L for P3 and N1P3 and with the chocolate odor for P2 and P3 (two-tailed unpaired t-test *P* < 0.05, MWA > MWoA)

According to the ERPs (Fig. 1), the amplitude of the CO_2_ response in the left nostril was significantly higher in MWA compared to MWoA for P3 and N1P3 (two-tailed unpaired t-test, *df* = 19, for P3: *P* = 0.013, 95% CI [-2.14, -0.25], Cohen’s D = -1.21; for N1P3: *P* = 0.038, 95% CI [-1.89, -0.06], Cohen’s D = -0.99). In addition, the amplitude of the chocolate response in the left nostril was significantly higher in MWA compared to MWoA for P2 and P3 (two-tailed unpaired t-test, *df* = 15, for P2:* P* = 0.035, 95% CI [-2.14, -0.08], Cohen’s D = -1.13; for P3: *P* = 0.023, 95% CI [-2.26, -0.16], Cohen’s D = -1.23). No significant effects were found on amplitudes for other peaks (P1, N1, P1N1, N1P2) and stimuli (CO_2_ in the right nostril, chocolate in the right nostril). No other effect was found on other peak latencies. A repeated measures ANOVA revealed significant effects: the stimulation type affected the amplitude of P2 (*df* = 1, *F* = 5.87, *P* = 0.036, η^2^ = 0.06, CO_2_ > chocolate). A general interaction between the stimulated side and the stimulation type was also found for P1 and N1 (P1: *df* = 1, *F* = 22.80, *P* < 0.001, η^2^ = 0.07, left > right for chocolate and right > left for CO_2_; N1: *df* = 1, *F* = 5.67, *P* = 0.039, η^2^ = 0.07, right > left for chocolate and left > right for CO_2_), which also depended on the aura status of the patients for P1 (*df* = 1, *F* = 5.88, *P* = 0.036, η^2^ = 0.02, MWA had higher amplitudes for chocolate on the left and for CO_2_ on the right side, while MWoA had higher chocolate and CO_2_ amplitudes on the right side). In other words independent of the patient group, the P1 and N1 amplitudes were higher for left-sided stimulations with chocolate compared to right-sided ones, and it was the other way around for stimulation with CO_2._ Finally, there was an interaction between the nostril side and the aura group for P2, P3 and N1P2 amplitudes (P2: *df* = 1, *F* = 7.32, *P* = 0.022, η^2^ = 0.04; P3: *df* = 1, *F* = 5.47, *P* = 0.041, η^2^ = 0.04; N1P2: *df* = 1, *F* = 6.41, *P* = 0.030, η^2^ = 0.03; for all peaks: left > right in MWA, right > left for MWoA): the left-sided amplitudes for P2 and N1P2 were higher than the right-sided stimulations in MWA and it was the opposite for MWoA. The corresponding post hoc tests were non-significant.

**Fig. 2:**
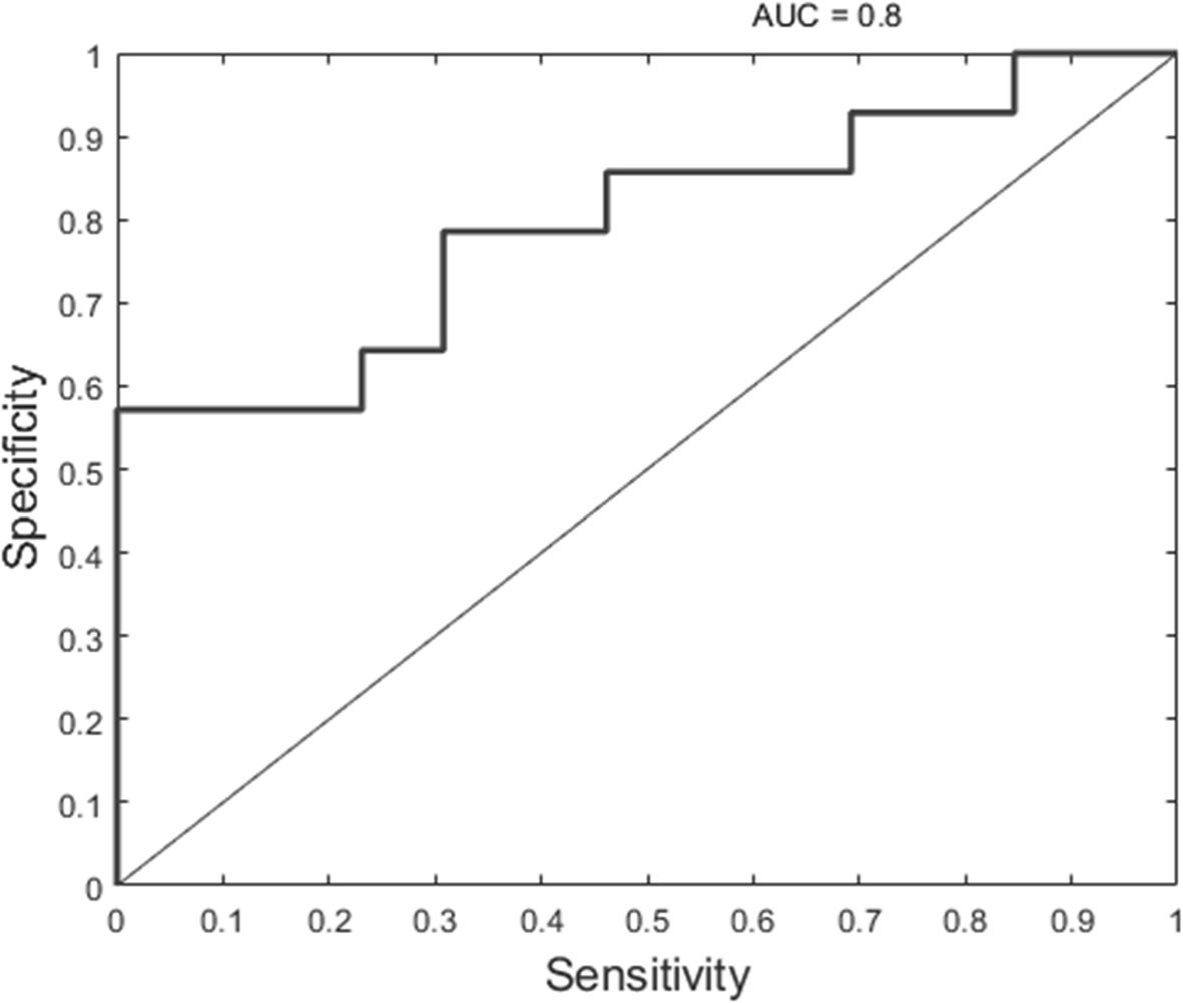
ROC curve and corresponding Area Under the Curve (AUC) for P3 amplitude for left-sided CO_2_. The model calculated the specificity and sensitivity of classification of P3 amplitudes between MWA and MWoA. This corresponded to Pz electrode signal

Among all the peak and peak-to-peak amplitudes, P3 amplitudes in response to CO_2_ in the left nostril elicited the best score in predicting the group affiliation with an AUC of 0.80, with *P* = 0.02 (Fig. 2). There was also a tendency for P2 amplitudes in response to left-sided CO_2_: AUC 0.71 and *P* = 0.09.

In addition, the P3 amplitudes for left-sided CO2, the P2 and P3 amplitudes for right-sided CO2 and the P1 amplitudes for left-sided chocolate stimulation were significantly higher for the patients who answered yes than the ones who answered no to the question “can odors trigger a migraine attack?” (two-tailed independent samples t-test, P3 CO2L: *t(18)* = 2.24, *P* = 0.038, Cohen’s D = 1.25; P2 CO2R: *t(17)* = 2.82, *P* = 0.012, Cohen’s D = 1.59; P3 CO2R: *t(17)* = 2.72, *P* = 0.015, Cohen’s D = 1.53; P1 ChocL: *t(15)* = 2.17, *P* = 0.047, Cohen’s D = 1.63). No differences of amplitudes were found between the patients who answered yes or no to the question “Are you sensitive to scents before/during the migraine attack”. The duration of migraine attacks had an impact on P2 amplitudes for left-sided CO2 stimulation: the longer the duration, the lower the amplitude (Spearman’s rho = -0.498, *P* = 0.035). An age-related effect was also found for right-sided trigeminal P1 (Spearman’s rho = 0.465, *P* = 0.045, increasing amplitude with age) and right-sided olfactory P2 (Pearson’s *r* = -0.490, *P* = 0.021, decreasing amplitude with age).

### Time–frequency analysis

**Fig. 3:**
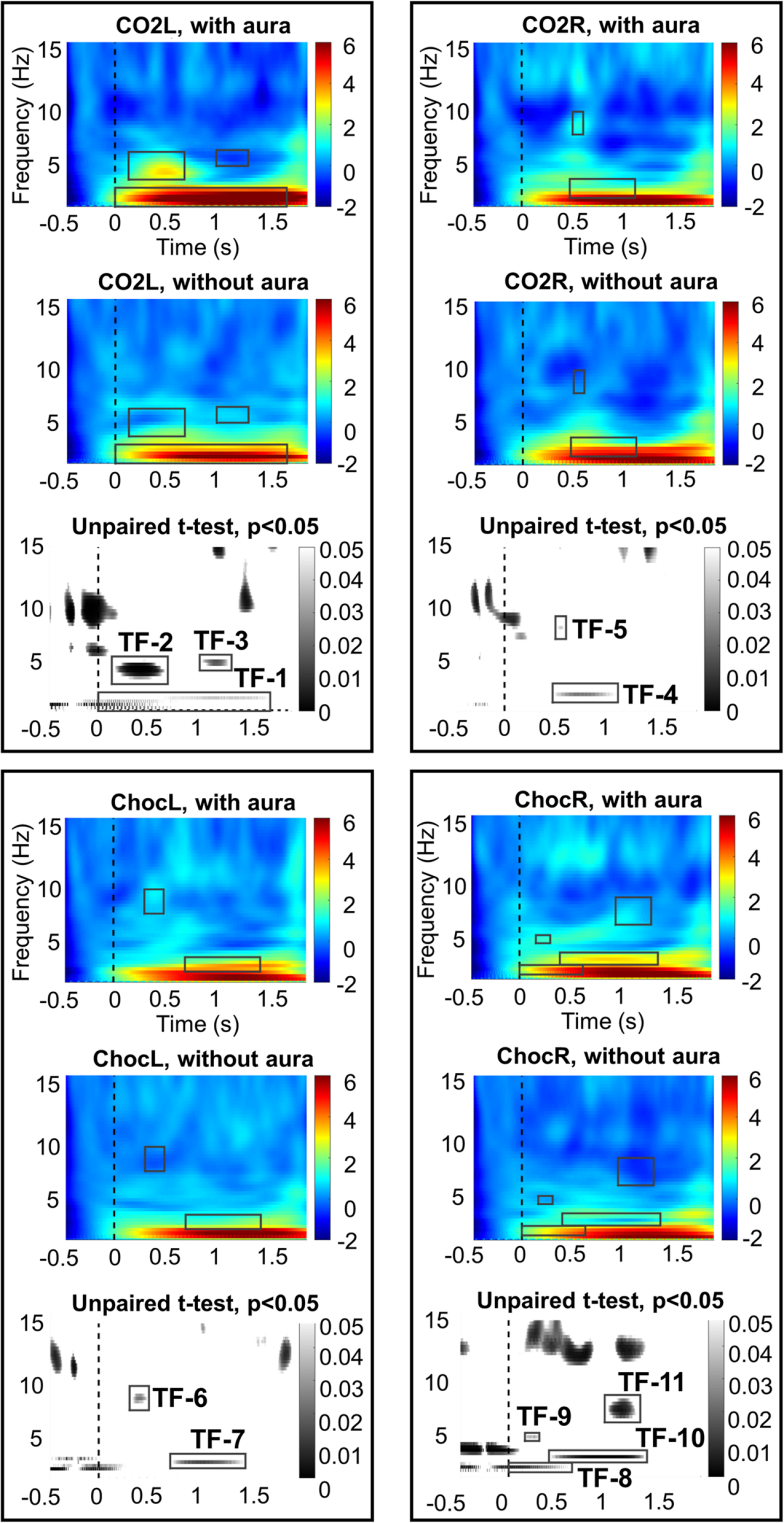
Time–frequency analysis (TFA) on ERPs. Significant differences between MWA and MWoA are marked by black rectangles: only frequencies in the lower range (below 8 Hz) are reported. These time–frequency windows are numbered with a practical aim for further description, i.e. TF-1…TF-11 (Table 2). Maps represent frequencies across time, time-point 0 being the stimulus onset (vertical dashed line). Color scales express the power (µV^2^/Hz) and are normalized across groups and stimuli. The data was taken from Pz electrode. Choc: Chocolate, L: left, R: right

The time frequency analysis revealed a significant difference between MWA and MWoA concerning brain responses to all stimulation types (unpaired t-test, *P* < 0.05), see Fig. 3.

The CO_2_ stimulation in the left nostril led to a stronger power in low frequencies (< 8 Hz) in MWA compared to MWoA (see Table 2 for details). On the contrary, in a later stage, MWoA had stronger power in low frequencies compared to MWA. The right-sided CO_2_ stimuli also induced power differences with higher power for MWA in the range of theta (theta: 4-8 Hz) frequencies, and stronger power for MWoA in the delta (delta: 0.1-4 Hz) frequency range. The left- and right-sided chocolate stimulations induced a stronger power in low frequencies (< 8 Hz) for MWA compared to MWoA (see the black rectangles in Fig. 4).

**Table 2:** Time–frequency windows in the low frequencies that are significantly different between MWA and MWoA

TF window	x limits (s)	y limits (Hz)	*P* min	T value	x_*P*min_ (s)	y_*P*min_ (Hz)	Power (MWA/MWoA)	Directionality
TF-1	0–1.78	0.3–1.99	0.004	-2.89	0.011	0.9	2.75/2.03	MWA > MWoA
TF-2	0.14–0.72	2.72–5.20	< 0.001	-3.68	0.436	4.2	2.43/0	MWA > MWoA
TF-3	1.05–1.38	3.94–5.36	0.016	2.41	1.221	4.8	-0.62/0.70	MWA < MWoA
TF-4	0.50–1.18	1.07–2.78	0.023	2.28	0.809	1.8	2.27/4.35	MWA < MWoA
TF-5	0.53–0.64	6.77–8.82	0.039	0.04	0.592	7.8	1.22/-0.11	MWA > MWoA
TF-6	0.32–0.52	6.45–8.63	0.023	-2.28	0.426	7.5	0.78/-0.30	MWA > MWoA
TF-7	0.74–1.52	1.25–2.55	0.010	-2.51	1.092	1.8	3.77/1.66	MWA > MWoA
TF-8	0–0.66	0.72–1.57	0.004	-2.84	0.039	1.2	2.49/1.90	MWA > MWoA
TF-9	0.17–0.32	3.53–4.26	0.03	-2.15	0.266	3.9	1.43/0.22	MWA > MWoA
TF10	0.42–1.44	1.60–2.70	< 0.001	-3.37	1.065	2.1	3.31/0.33	MWA > MWoA
TF-11	1–1.37	5.20–7.66	0.004	-2.92	1.180	6.3	0.77/-0.63	MWA > MWoA

These time–frequency windows are numbered for practical aim (i.e. TF-1…TF-11) and refer to the Fig. 3. The limits of the windows are described (x and y limits); the lower *P* values (*P* min) of these windows were extracted with corresponding T values, x and y (x_*P*min_ and y_*P*min_), power value in each patient group and t-test directionality

### Source localization results

The right-sided stimulation with CO_2_ involved higher neural activity in MWA compared to MWoA during four main time-courses (unpaired t-test, *P* < 0.05, subtracting groups: MWA-MWoA, positive t-values) (for more details, see Fig. 4 from Additional file 5). This was in the early stage of the response (248–289 ms post-stimulus) in the left fusiform gyrus (Fus) and the left precuneus (Prec). It was secondly in the right postcentral gyrus (A1), the right caudal inferior parietal lobule, and the left putamen (353–438 ms post-stimulus), the left superior parietal lobule, right pons. The third time period corresponded to 482–738 ms post-stimulus and involved the right precentral gyrus (brodmann 4). Finally, in a later stage of the response (978–1051 ms post-stimulus), MWA involved more their right calcarine sulcus (primary visual cortex V1, hOc1) and premotor cortex (BA6).

The left-sided stimulation with chocolate involved lower neural activity in MWA than MWoA firstly in the bilateral paracentral lobule, the precentral gyrus, the left superior parietal lobule, the left amygdala, the left parahippocampal gyrus, the left culmen of the cerebellum, the left pons. This happened in the middle phase post-stimulus, i.e. around 357–441 ms post-stimulus (unpaired t-test, *P* < 0.05, subtracting groups: MWA-MWoA, negative t-values) (for more details, see Fig. 5 from Additional file 5). In addition, in a later stage of the response (482-738 ms post-stimulus), MWA presented lower neural activity than MWoA in the right middle frontal gyrus, the right lentiform nucleus of the putamen and the right brainstem.

The right-sided stimulation with chocolate involved lower neural activity in MWA than MWoA in the left lateral globus pallidus, the left lentiform nucleus, the left thalamus, left amygdala, the left parahippocampal gyrus, and the left pons. This happened in the middle phase post-stimulus, i.e. around 570–724 ms post-stimulus (unpaired t-test, *P* < 0.05, subtracting groups: MWA-MWoA, negative t-values) (for more details, see Fig. 6 from Additional file 5).

## Discussion

To assess whether olfactory and trigeminal stimuli are differentially processed in MWA and MWoA, event-related potentials have been compared for both groups.

### Hypersensitivity to nociceptive (CO_2_) trigeminal stimuli in MWA

MWA showed stronger responses to left-sided trigeminal stimulations, P3 amplitudes constituted a good discriminant between groups. Previous studies also showed hypersensitivity to different types of noxious stimuli in migraine patients [15, 45–47], which could be partly driven by MWA.

Theta oscillations were mostly increased in MWA during CO_2_ responses. As time–frequency analysis deciphers non-phase locked activity, it is not surprising that these additional group differences appear. Intranasal trigeminal stimulation usually leads to an increase of low frequency oscillations [27]. Thus, the theta band pattern might confirm MWA hypersensitivity to trigeminal stimuli. Expectations regarding the trigeminal stimulus may explain differences in delta oscillations between nostril sides. Indeed, this was not counterbalanced across patients, the left nostril being always stimulated first. Thus, the over-sensitivity to CO2L might have been partly compensated during CO2R by some improvement in pain expectation. Other explanations may be the impact of CO_2_ stimulation side on the brain structures involved [48], and a strong lateralization of the pain processing on the right hemisphere [49–51].

The right-sided trigeminal response led to higher neural activity in MWA for pain network structures [49, 52, 53] that participate to pain intensity perception, and the postcentral gyrus is one of the sources for pain ERP [52]. A similar result is also suggested [46] that while comparing MWA with healthy controls, a painful heat stimulus induced hyperactivity of the somatosensory cortex. Other studies in a mixed migraine population report a hypo-activity [54, 55]. This discrepancy points at a differential trigeminal processing between MWA and MWoA and the importance of studying them separately.

The reported structures were differentially activated during a time period including mostly cognitive aspects [39], some are involved in catastrophization, expectations [49, 52, 53], learning and attention towards pain [56, 57]. In healthy subjects, intranasal trigeminal stimulation involves visual-associative areas [58]. Here, the right precentral gyrus and the right calcarine sulcus were more recruited in MWA. Participants were requested to avoid blinking as much as possible, however, the trigeminal nerve includes an ophthalmic branch leading intranasal trigeminal stimuli to trigger blinking reflexes. Although only epochs clean of eye artefacts were considered, MWA may have engaged more a visual-associative network to refrain blinking.

Altogether, these results suggest hypersensitivity and attentional bias toward painful stimuli in MWA with a possible increment in pain anticipation and catastrophizing.

### Differential olfactory processing between MWA and MWoA

MWA had a lower olfactory detection ability. In line with this, in a larger population, migraine patients have higher olfactory perception thresholds [59], MWA present a lower olfactory sensitivity compared to MWoA, but a better odor discrimination [60] and a similar identification ability [61].

P2 and P3 peaks for left-sided olfactory stimulation were larger for MWA, they had more low-frequency oscillations for both sides. An olfactory ERP would normally appear in the low frequencies (< 15 Hz) in healthy subjects [27]. Another study showed decreased ERP amplitudes to olfactory stimuli for migraine patients compared to healthy subjects [15]. Altogether, these results suggest an impairment of the olfactory brain processing in MWA, with a higher amount of low-frequency oscillations and larger cognitive peaks that might reflect their lower sensitivity and higher discrimination.

The response to olfactory stimulations resulted in a lower neural activity in MWA in several brain regions, and most of these structures do not belong to the primary olfactory cortex. Some appear to be functionally connected to the frontal or temporal part of the piriform cortex and might be involved in planning motor actions in response to odors and sneezing [62]. In addition, some are involved in cognitive aspects of olfaction: learning and memory [63], familiarity [64], attention and pleasantness judgements [63, 65, 66]. These differences were present during a time period referring usually to cognitive aspects of the processing [39].

Altogether, MWA seem to have a bigger deficit in engaging secondary olfactory-related structures, leading to distorted attention and judgements towards odors.

### Overlap between trigeminal and olfactory systems

All stimuli elicited differential brain oscillations between MWA and MWoA. In addition, patients who are considered osmophobic when answering yes at “can odors trigger a migraine attack?” had some higher peak amplitudes for olfactory and trigeminal stimuli.

The interaction between the olfactory and the trigeminal system might explain these results. In daily life, most odors are a mixture of olfactory and trigeminal compounds [25], activating both pathways [67] and the pain network [68, 69]. On one hand, patients with migraine exhibit an olfactory bulb atrophy, especially osmophobic patients with a left-sided pronounced deficit [70, 71]. Additionally, MWA are found to be more prone to osmophobia during attacks [7, 72] and odors are more offensive to them [7] compared to MWoA; although some studies suggest the opposite [10, 73]. Hence, it is legitimate to wonder whether the olfactory bulb atrophy is more common in MWA or MWoA.

Some trigeminal ganglion cells send axons to the olfactory bulb [74] and the theta/delta frequency bands reflect activity from the spinal trigeminal nucleus [58]. Thus, it is likely that the hypersensitivity to trigeminal stimuli found in MWA originates from the spinal trigeminal nucleus and modifies the olfactory bulb oscillations. However, further research is necessary based on the impact of the olfactory bulb oscillations on further structures in the olfactory pathway in rodents [75]. This could explain alternatively differences in olfactory oscillations between groups.

Further central processing may also be involved [17, 76]. Migraine has been suggested as a cerebral “connectopathy” with increased activity in the so-called neurolimbic-pain network and higher connectivity between cortical nodes involved in pain in MWoA [77], originating partly from limbic structures and the thalamus [54, 78–80]. Thus, some differences in olfactory and trigeminal processing between groups might originate from differential brain oscillations abnormality in these networks.

We hypothesize that for MWA, the olfactory aspect of bimodal odors would not be properly integrated which 1) would lead to, or 2) would be due to an exaggerated trigeminal processing. This could possibly arise from abnormal oscillations at the level of one of the overlapping brain structures.

### Limitations and perspectives

The present sample size was relatively small. However, this was based on previous reports with EEG assessments of odor stimulations [15, 27–30]. The source reconstruction results presented here are uncorrected. Secondly, the differences between groups found for the odor and trigeminal stimuli were not consistent between sides of stimulation. Whether our results are due to a lateralized olfactory bulb atrophy is a topic for future research. In addition, data were collected during the Covid-19 pandemic. It has been shown that Covid-19 can induce temporary, or sometimes complete smell loss [81]. In addition, during lockdown, smells were precipitating factors of migraine attacks even more than before the pandemic [82, 83], and higher post-infection olfactory symptoms were found in migraine Covid-19 survivors as compared to non-migraine survivors [84]. None of the patients included here had Covid-19, or had recovered from Covid-19 in the two weeks prior to testing. However, the past infection status was not recorded. Although all the patients were normosmics, having this information would have been useful, especially to assess whether some differences between MWA and MWoA occur in this regard. Due to methodological constraints, the order of stimulation delivery was not counterbalanced. If not handled carefully, repeated odorous or trigeminal exposure can induce habituation, even in patients with migraine [85]. Consequently, ERP amplitudes can decrease over time. This is however unlikely here as the nostril side had an opposite effect on the results depending on the stimulation type. Another limitation is that for ethical reasons, the patients were allowed to take acute medication, but also migraine prophylactic treatment (for more detail, see Additional file 4). We did not actively ask them if they took some acute medication on the day of the appointment. It is however unlikely as none of the patients who took part in the study had migraine attacks the day before or the day after the EEG assessment. The two experimenters collecting data had also access to the patient’s status (MWA or MWoA). Finally, the present study did not include a control group, however previous studies have shown higher olfactory ERP responses and lower trigeminal ERP responses in healthy participants as compared with migraine patients [15]. This provides some insights regarding how healthy controls process these stimuli.

## Conclusions

During trigeminal nociceptive stimulation, depending on the nostril side, patients with migraine with aura show pronounced hyperactivity in cerebral pain network structures as compared to patients without. Different olfactory oscillations are also found, with differences of activation patterns in structures especially involved in pain-related, visual related and secondary olfactory structures. The overlap between nociception and olfaction provides a clue to understand why patients with migraine with aura appear more clinically impaired than patients without, as some structures shared by both sensory systems show differential activity patterns.

## Supplementary Information

**Additional file 1:** Number of epochs accepted for averaging. Description of data: the number of epochs selected for the grand average for each condition

**Additional file 2:** Information about latencies out of usual boundaries for chemosensory ERP. Description of data: the mean and standard deviations of the difference between the measured latency and the closer latency limit set for usual chemosensory ERP.

**Additional file 3:** Number of participants with missing data. Description of data: number of participants with missing ERP.

**Additional file 4:** Distribution of medication among patients. Description of data: number of patients taking acute or prophylactic medication and some details about medications.

**Additional file 5:** Differential source localization results between MWA and MWoA. Description of data: slice projections and 3D glass views of differential neural activity between MWA and MWoA for CO2R, ChocL and ChocR.

## Data Availability

The datasets used and/or analysed during the current study are available from the corresponding author on reasonable request.

## Abbreviations

BOLD: Blood Oxygen Level Dependent
TDI: Threshold, Discrimination, Idenditication
ERPs: Event-Related Potentials
MWA: Patients with migraine with Aura
MWoA: Patients with migraine without Aura
rCBF: Regional Cerebral Blood Flow
ROI: Region of Interest
SNR: Signal to Noise Ratio
