# Supplementary material for: Migraine with aura: less control over pain and fragrances?
Source: J Headache Pain. 2023 May 17;24(1):55. doi: 10.1186/s10194-023-01592-3 (PMC10189721; doi:10.1186/s10194-023-01592-3)
Supplement: Supplementary file 1 — Additional file 1: Number of epochs accepted for averaging. Description of data: the number of epochs selected for the grand average for each condition [file 10194_2023_1592_MOESM1_ESM.docx]

**Additional file 1: Number of epochs accepted for averaging**

CO_2_L aura: 205, CO_2_L without aura: 238, CO_2_R aura: 189, CO_2_R without aura: 227, ChocL aura: 213, ChocL without aura: 271, ChocR aura: 231, ChocR without aura: 277.
