# Supplementary material for: Migraine with aura: less control over pain and fragrances?
Source: J Headache Pain. 2023 May 17;24(1):55. doi: 10.1186/s10194-023-01592-3 (PMC10189721; doi:10.1186/s10194-023-01592-3)
Supplement: Supplementary file 2 — Additional file 2: Information about latencies out of usual boundaries for chemosensory ERP. Description of data: the mean and standard deviations of the difference between the measured latency and the closer latency limit set for usual chemosensory ERP. [file 10194_2023_1592_MOESM2_ESM.docx]

**Additional file 2: Information about latencies out of usual boundaries for chemosensory ERP**

Here are the mean and standard deviations of the difference between the measured latency and the closer latency limit set for usual chemosensory ERP.

Lower boundaries:

P3: mean 60ms, SD 83.2ms.

Higher boundaries:

P1: mean 219.5ms, SD 183.1ms; N1: mean 164.5ms, SD 147.8ms; P2: mean 141ms, SD 170.4ms; P3: mean 249.6ms, SD 225.8ms.
