# Supplementary material for: Migraine with aura: less control over pain and fragrances?
Source: J Headache Pain. 2023 May 17;24(1):55. doi: 10.1186/s10194-023-01592-3 (PMC10189721; doi:10.1186/s10194-023-01592-3)
Supplement: Supplementary file 3 — Additional file 3: Number of participants with missing data. Description of data: number of participants with missing ERP. [file 10194_2023_1592_MOESM3_ESM.docx]

**Additional file 3: Number of participants with missing data**

CO_2_L aura: 1, CO_2_L without aura: 6, CO_2_R aura: 5, CO_2_R without aura: 4, ChocL aura: 6, ChocL without aura: 6, ChocR aura: 3, ChocR without aura: 3.
