# Supplementary material for: Migraine with aura: less control over pain and fragrances?
Source: J Headache Pain. 2023 May 17;24(1):55. doi: 10.1186/s10194-023-01592-3 (PMC10189721; doi:10.1186/s10194-023-01592-3)
Supplement: Supplementary file 4 — Additional file 4: Distribution of medication among patients. Description of data: number of patients taking acute or prophylactic medication and some details about medications. [file 10194_2023_1592_MOESM4_ESM.docx]

**Additional file 4: Distribution of medication among patients**

During the month prior to investigation, MWA took acute medication in average during 2.89 ± 2.09 days with a successful action over 2.86 ± 2.41 days, while MWoA took it during 2.80 ± 2.30 days with a successful action over a period of 2.42 ± 2.15 days. The distribution of medication among patients was the following:

**Prophylactic medication:**

BetaBlocker: 2

Topiramat: 0

Flunazinin: 1

Amitryptilin: 0

Others: 4

None: 23

**Acute medication:**

Ibuprofen: 13

Paracetamol: 5

Novalgin: 1

Triptane: 13

ASS: 3

Vomex: 0

Peppermint oil: 0

Naproxen: 1

Others: 5

None: 2
