# Supplementary material for: Migraine with aura: less control over pain and fragrances?
Source: J Headache Pain. 2023 May 17;24(1):55. doi: 10.1186/s10194-023-01592-3 (PMC10189721; doi:10.1186/s10194-023-01592-3)
Supplement: Supplementary file 5 — Additional file 5: Differential source localization results between MWA and MWoA. Description of data: slice projections and 3D glass views of differential neural activity between MWA and MWoA for CO2R, ChocL and ChocR. [file 10194_2023_1592_MOESM5_ESM.docx]

**Additional file 5: Differential source localization results between MWA and MWoA**


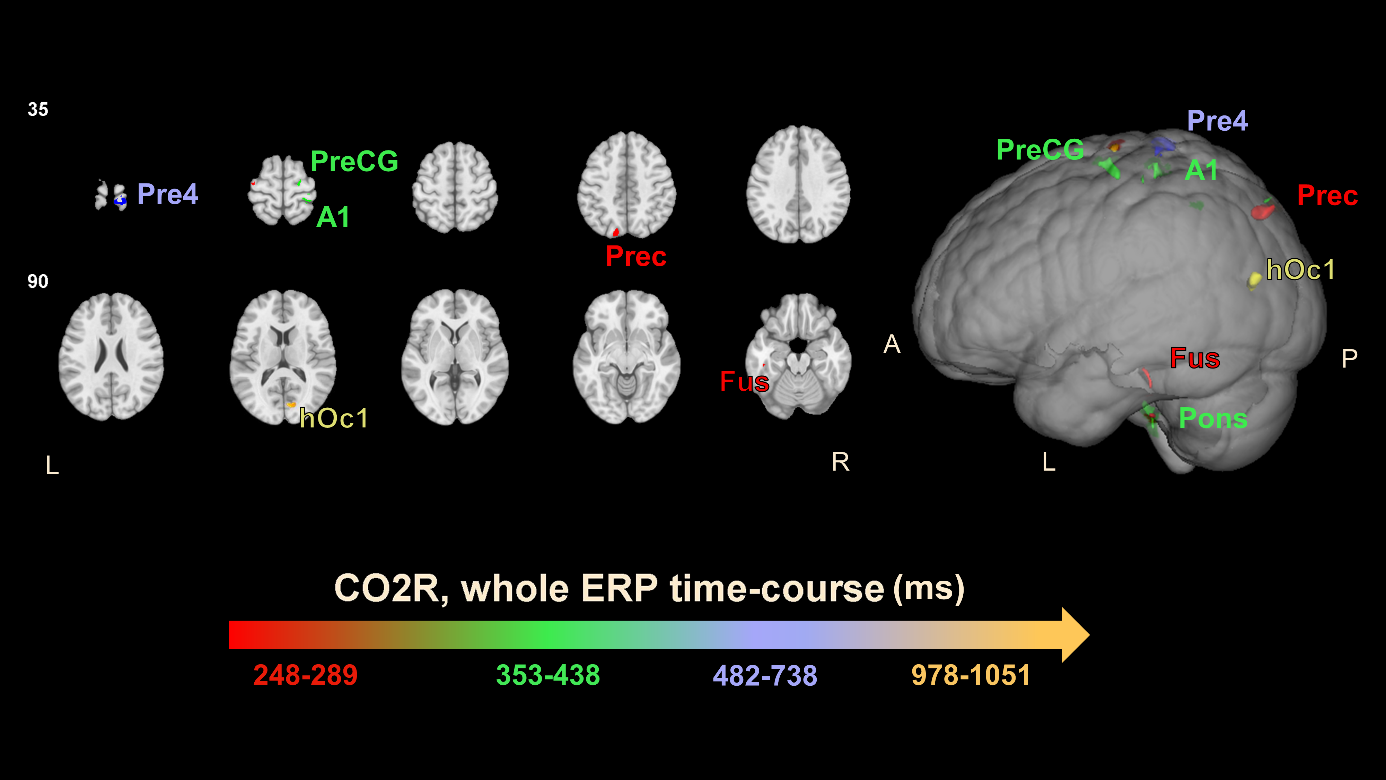


**Fig. 4 Differential source localization for CO_2_R between MWA and MWoA in four post-stimulus time-ranges.** The left panel shows the source reconstruction on slices, the right panel presents the same source reconstruction in a 3D glass view. Slice levels are reported on the left side of the rows. LAURA algorithm was used, and unpaired t-test was performed on the whole epoch time-course. Results were considered as valid when at least 16 consecutive time-frames (around 30 ms) were significant (p < 0.05), the mean over the corresponding period of time is reported. The data are uncorrected. L: left, R: right, P: posterior, A: anterior, Pre4: precentral gyrus, A1: postcentral gyrus, PreCG: precentral gyrus, Prec: precuneus, hOc1: calcarine sulcus, Fus: fusiform gyrus.


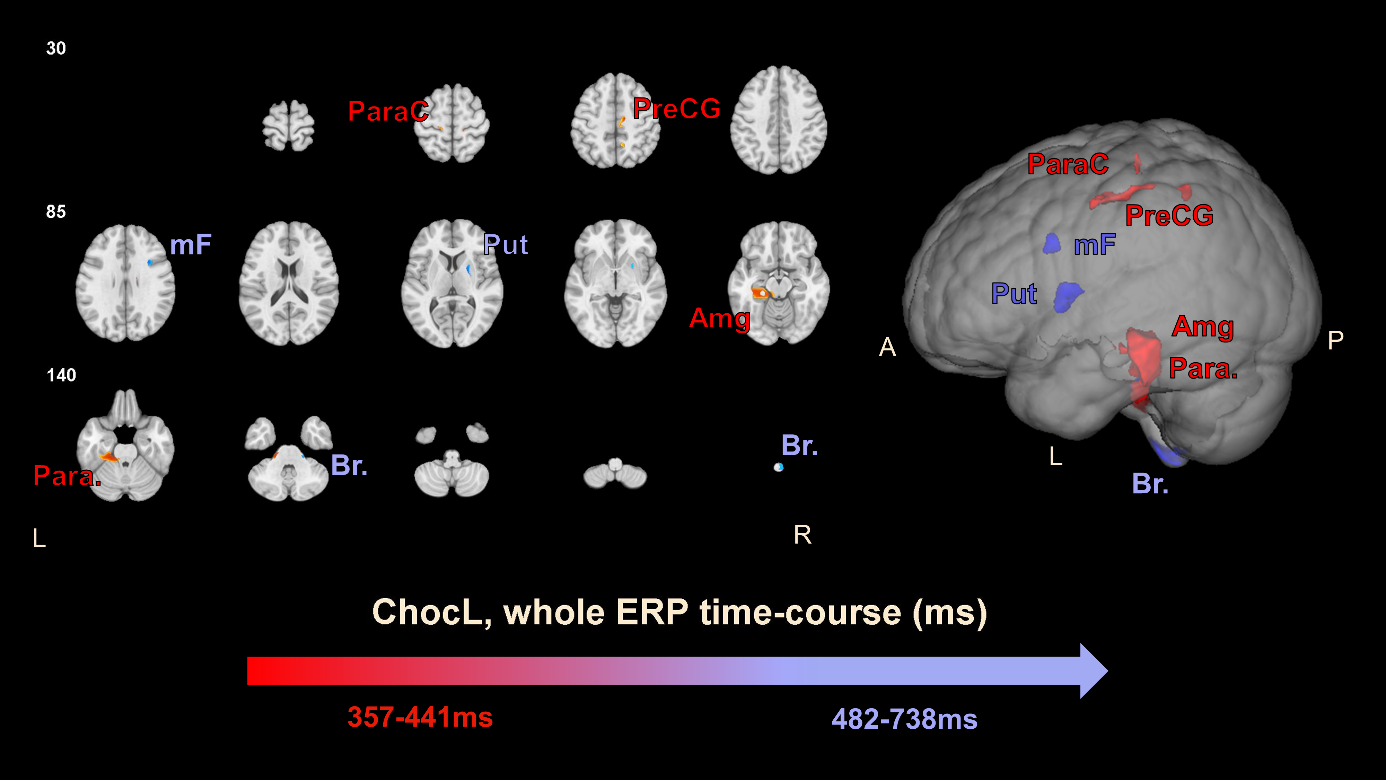


**Fig. 5 Differential source localization for ChocL between MWA and MWoA in two post-stimulus time-ranges.** The left panel shows the source reconstruction on slices, the right panel presents the same source reconstruction in a 3D glass view. Slice levels are reported on the left side of the rows. LAURA algorithm was used, and unpaired t-test was performed on the whole epoch time-course. Results were considered as valid when at least 16 consecutive time-frames (around 30 ms) were significant, the mean over the corresponding period of time is reported. The data are uncorrected. L: left, R: right, P: posterior, A: anterior, ChocL: left sided chocolate odor. ParaC: paracentral lobule, PreCG: precentral gyrus, mF: middle frontal gyrus, Put: lentiform nucleus of the putamen, Amg: amygdala, Para: parahippocampal gyrus, Br.: brainstem.

**
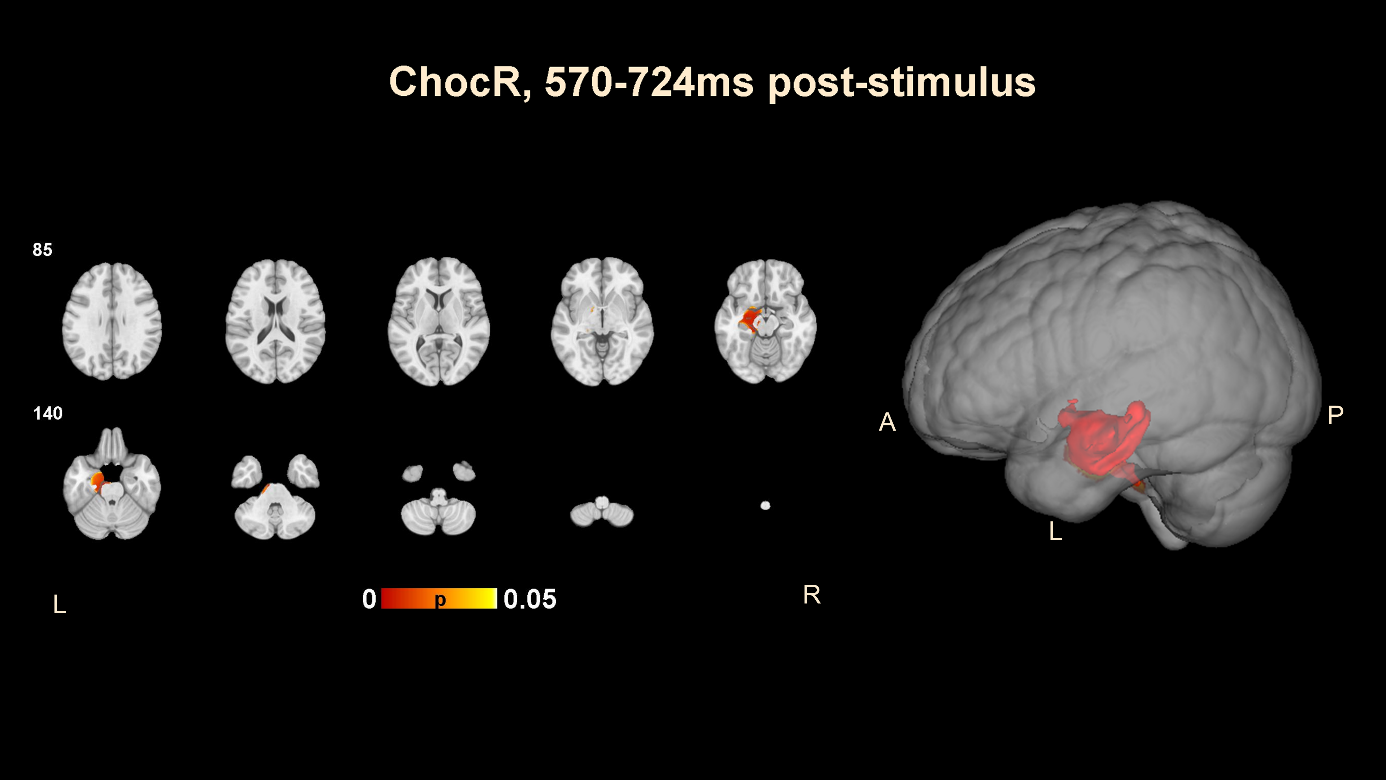
Fig. 6 Differential source localization for ChocR between MWA and MWoA in the 570-724 ms post-stimulus time-range.** The left panel shows the source reconstruction on slices, the right panel presents the same source reconstruction in a 3D glass view. Slice levels are reported on the left side of the rows. LAURA algorithm was used, and unpaired t-test was performed on the whole epoch time-course. Results were considered as valid when at least 16 consecutive time-frames (around 30 ms) were significant, the mean over the corresponding period of time is reported. The data are uncorrected. L: left, R: right, P: posterior, A: anterior, ChocR: right sided chocolate odor.
